# Altered structure of the vestibular cortex in patients with vestibular migraine

**DOI:** 10.1002/brb3.1572

**Published:** 2020-03-10

**Authors:** Xia Zhe, Jie Gao, Li Chen, Dongsheng Zhang, Min Tang, Xuejiao Yan, Fuxia Bai, Xin Zhang, Ze Zou, Weibo Chen, Xiaoyan Lei, Xiaoling Zhang

**Affiliations:** ^1^ Department of MRI Diagnosis Shaanxi Provincial People's Hospital Xi'an China; ^2^ Department of Neurology Shaanxi Provincial People's Hospital Xi'an China; ^3^ Philips Healthcare Shanghai China

**Keywords:** gray matter volume, migraine, vertigo, vestibular cortex, vestibular migraine, voxel‐based morphometry

## Abstract

**Introduction:**

Previous voxel‐based morphometry (VBM) studies have revealed changes in brain structure in patients with vestibular migraine (VM); these findings have improved the present understanding of pathophysiology. Few other studies have assessed the association between structural changes and the severity of dizziness in VM. This study aimed to examine the structural changes and cortical morphometric features associated with migraine and vertigo attacks in patients with VM.

**Methods:**

Twenty patients with VM and 20 healthy normal volunteers were scanned on a 3‐tesla MRI scanner. The gray matter volume (GMV) was estimated using the automated Computational Anatomy Toolbox (CAT12). The relationship between clinical parameters and morphometric abnormalities was also analyzed in VM.

**Results:**

Compared with controls, VM patients have decreased GMV in the prefrontal cortex (PFC), posterior insula–operculum regions, inferior parietal gyrus, and supramarginal gyrus. Moreover, patient scores on the Dizziness Handicap Inventory (DHI) score showed a negative correlation with GMV in the posterior insula–operculum regions.

**Conclusion:**

These findings demonstrated abnormality in the central vestibular cortex and correlations between dizziness severity and GMV in core regions of the vestibular cortex of VM patients, suggesting a pathophysiological role of these core vestibular regions in VM patients.

## Graphical abstract

This study employs computational anatomy toolbox (CAT12) to assess the patterns of regional gray matter volumetric (GMV) abnormalities in patients with vestibular migraine (VM). Our results demonstrated that structural multiple vestibular cortex deficits in VM patients, especially abnormalities in posterior insula–operculum region of vestibular cortical core region, suggesting that these areas were involved in the pathophysiological of VM.

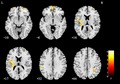

## 1 INTRODUCTION

Although there is still some debate on the status of vestibular migraine (VM) as a distinct clinical entity (Phillips, Longridge, Mallinson, & Robinson, 2010; von Brevern et al., 2011), diagnostic criteria for this criterion have been proposed by the Committee for the Classification of Vestibular Disorders of the Barany Society and the Headache Classification Committee of the International Headache Society (IHS, 2013; Lempert et al., 2012). VM is characterized by vestibular symptoms, such as recurrent episodes of vertigo and migrainous symptoms including headache, photophobia, and phonophobia (Dieterich, Obermann, & Celebisoy, 2016; Furman, Marcus, & Balaban, 2013). Epidemiological studies have reported a lifetime VM prevalence of 1% and a 1‐year prevalence of 0.89% in the general population (Hochman, Preter, Neuhauser, & Lempert, 2001; Neuhauser et al., 2006). Women suffer VM twice to three times as frequently as men (Lempert & Neuhauser, 2009). Recurrent vertigo and migraine headaches impose a significant burden on the individual as well as society, resulting in productivity loss and even disability.

Gray matter volume (GMV) changes have been detected in several other vestibular disorders, such as vestibular neuritis (VN) and persistent postural perceptual dizziness (PPPD). Some studies have described structural alterations in patients with VN, which may be related to central vestibular compensation. Eulenberg et al. found GMV decreases in the superior temporal gyrus (STG), posterior hippocampus, and superior frontal gyrus as well as increases in the superior, inferior and middle temporal gyri (MT/V5) and the gracile nucleus (Eulenburg, Stoeter, & Dieterich, 2010). Helmchen et al. (2009) detected GMV increases primarily in the STG, cingulate cortex, MT/V5, cerebellum, insula, inferior parietal lobe as well as a GMV decrease in the midline pontomedullary junction. Similar changes were detected in recent studies on patients with PPPD. Wurthmann et al. (2017) found a GMV decrease in the temporal cortex, cingulate cortex, precentral gyrus, hippocampus, dorsolateral prefrontal cortex (PFC), caudate nucleus, and cerebellum. These multisensory vestibular cortical areas are involved in central vestibular processing and compensation. However, there have been only a few studies on structural imaging techniques to assess structural changes in patients with VM previously. Among the cortical regions involved in multisensory vestibular control and central vestibular compensation, those studies have shown GMV loss in the MT/V5 and the middle cingulate, dorsolateral prefrontal, insular, parietal and occipital cortices (Obermann et al., 2014). In contrast, some studies on voxel‐based morphometry (VBM) have shown that the GMV is increased regionally in the frontal, occipital, and angular regions in VM patients compared to controls (Messina et al., 2017; Wang et al., 2019). Furthermore, correlation analyses have suggested that VM may induce cumulative effects on the structure of the brain. These analyses show a reduction in GMV during pain, and the vestibular processing areas may be associated with longer disease duration and increased headache severity (Obermann et al., 2014). Based on the above‐mentioned structural findings, recurrent VM attacks ultimately result in morphological alterations in the brain areas that are involved in pain and vestibular processing. However, these studies have some limitations. The patient clinical parameters examined in such studies were mainly focused on the pain intensity of migraine attacks and clinical measures of headache‐related disability via the Headache Impact Test‐6 (HIT‐6), Migraine Disability Assessment (MIDAS), and visual analogue scale (VAS). However, these studies did not consider any questionnaires measuring dizziness‐related symptoms (Obermann et al., 2014). According to previous studies, many VM patients suffer from balance problems, including vertigo attacks (Akdal, Baykan, et al., 2015; Akdal, Ozge, & Ergor, 2013, 2015; Cho et al., 2015; Dieterich et al., 2016).

Previous structural and functional imaging has suggested that the parieto‐insular vestibular cortex (PIVC), located in the posterior insula, retroinsular region, and parietal operculum, is the core of the human vestibular cortex and plays a key role in vestibular processing (Dieterich & Brandt, 2018; Frank & Greenlee, 2018; Lopez, Blanke, & Mast, 2012; Ventre‐Dominey, 2014; zu Eulenburg, Caspers, Roski, & Eickhoff, 2012). Therefore, in this study, we hypothesized that GMV might change in the PIVC regions of patients with VM compared to normal controls (NCs) and that these changes might be correlated with patients' clinical parameters. We used the Computational Anatomy Toolbox (CAT12) to assess whether GMV changed in patients with VM. Next, a correlation analysis was performed to identify previously unreported structural brain changes associated with migraine and dizziness attacks. To avoid limitations from previous studies, our study not only considered migraine‐related clinical questionnaires but also incorporated a dizziness‐related clinical questionnaire, the Dizziness Handicap Inventory (DHI), to assess the severity of this symptom in patients with VM.

## 2 MATERIALS AND METHODS

### 2.1 Subjects

Twenty right‐handed patients with VM were recruited from a vertigo and dizziness outpatient service center of the Shaanxi Provincial People's Hospital in China between January 2016 and June 2018. All patients were diagnosed with VM according to the diagnostic criteria of the Headache Classification Committee of the International Headache Society (International Classification of Headache Disorders [ICHD]‐3 beta, appendix) and the Committee for the Classification of Vestibular Disorders of the Barany Society (Headache Classification Committee of the International Headache Society, 2013; Lempert et al., 2012). VM patients with neurologic, psychiatric, audiovestibular, or systemic disorders were excluded. MRI scans were performed on day 3–7 after a VM attack, and all patients were required to be free of migraines and vertigo on the experimental day. All patients underwent a routine neurologic and neuro‐otological examination. No peripheral vestibular dysfunction was found in videonystagmography (VNG) recordings. Demographic data were collected from the patients in a face‐to‐face interview with a standardized questionnaire and questions. Patients rated the pain intensity of migraine attacks using the VAS (0 = no pain; 10 = worst possible pain) and additionally completed the MIDAS, HIT‐6 and DHI (Balci, Senyuva, & Akdal, 2018; Sauro et al., 2010). Four patients with VM were on migraine‐preventive medications (e.g., beta‐blockers). Additionally, six patients used nonsteroidal analgesics for attack treatment. Most of the investigated patients (*n* = 10) did not take any medication regularly.

Twenty age‐, sex‐, education‐, and handedness‐matched (right‐handed) NCs from the community with no history of migraine; chronic pain; previous VN; Meniere's disease; secondary somatoform vertigo; substance abuse; neurologic, mental, or systemic disorders; ischemic or hemorrhagic stroke; or severe head trauma were included. None of these subjects showed structural abnormalities or visible T2‐weighted hyperintensities in deep white matter on MRI examination. This study was approved by the Ethics Committee of the Shaanxi Provincial People's Hospital. All participants voluntarily provided written informed consent forms before entering the study.

### 2.2 Imaging data acquisition

The structural data were acquired on a 3.0 T Philips Ingenia scanner using a 16‐element phased‐array with only a head coil. A high‐resolution 3D magnetization‐prepared rapid‐acquisition gradient echo (MPRAGE) T1‐weighted sequence covering the whole brain (332 sagittal slices) was collected. The acquisition parameters were as follows: repetition time (TR): 1,900 ms; echo time (TE): 2.26 ms; inversion time (TI): 900 ms; flip angle: 9°; matrix: 256 × 256; field of view: 220 mm; and 1.00 mm slice thickness with no interslice gap.

### 2.3 Image processing

Structural scans were analyzed using CAT12 (http://dbm.neuro.unijena.de/cat) for SPM12 in MATLAB R2014b (The MathWorks, Inc.). CAT12 is one of the most important neuroimaging analysis techniques used to assess structural differences in regional gray volume (Besteher et al., 2017). Moreover, CAT12 can avoid operational bias in the selection of brain regions and in automated measurement of the whole brain. This toolbox includes bias‐field and noise removal; skull stripping; segmentation into gray matter, white matter, and cerebrospinal fluid; and, finally, normalization to MNI space using diffeomorphic anatomical registration using exponentiated Lie algebra (DARTEL) to a 1.5 mm isotropic adult template provided by the CAT12 toolbox. The resulting images were checked for homogeneity. As all the images had high correlation values (>0.85), none of the images had to be discarded. Finally, the gray matter images were smoothened using a Gaussian kernel with a full width at half maximum (FWHM) of 8 mm.

### 2.4 Statistical analysis

The group differences in demographic variables were examined by using independent *t* tests and analysis of covariance (ANCOVA) in SPSS 22.0. GMV was compared between VM patients and NCs using two‐sample *t* tests in SPM12 with patients' age, sex, and total intracranial volume (TIV) as covariates. The results were assessed at a threshold of *p* < .05 (false discovery rate [FDR] corrected) with a minimum cluster size set of 100 voxels. Thereafter, the statistically significant brain regions of the VM group were extracted as regions of interest (ROIs), and their correlation with patients' clinical parameters (including the severity of the headache attacks, disease duration [month], number of days per month with headaches [*n*], MIDAS, HIT‐6, and DHI) was analyzed using Pearson's partial correlation analysis in SPSS 22.0. Age and gender were controlling for as covariates. The significance threshold was set at *p* < .05.

## 3 RESULTS

### 3.1 Clinical data

The clinical and demographic characteristics of the VM and NC groups are presented in Table 1. There were no significant differences in age, sex, or years of education between VM patients and NCs (*p* > .05; Table 1).

**Table 1:** Demographic and clinical characteristics of the participants

Characteristics	VM (*n* = 20) Mean ± *SD*	NC (*n* = 20) Mean ± *SD*	*p*‐value
Gender (female/male)	18/2	17/3	.64
Age (years)	38.60 ± 11.10	37.60 ± 11.84	.78
Education (years)	13.75 ± 85	14.45 ± 85	.36
Disease duration (months)	78.00 ± 75.19		
Headache frequency (months)	6.90 ± 5.32		
VAS	5.35 ± 1.84		
MIDAS	56.05 ± 50.79		
HIT‐6	58.20 ± 7.15		
DHI	47.70 ± 11.11		

Abbreviations: DHI, Dizziness Handicap Inventory; HIT‐6, Headache Impact Test‐6; MIDAS, Migraine Disability Assessment Scale; NC, normal control; VAS, verbal rating scale (0 = no pain; 10 = worst possible pain); VM, vestibular migraine.

### 3.2 VBM results

**Figure 1:**
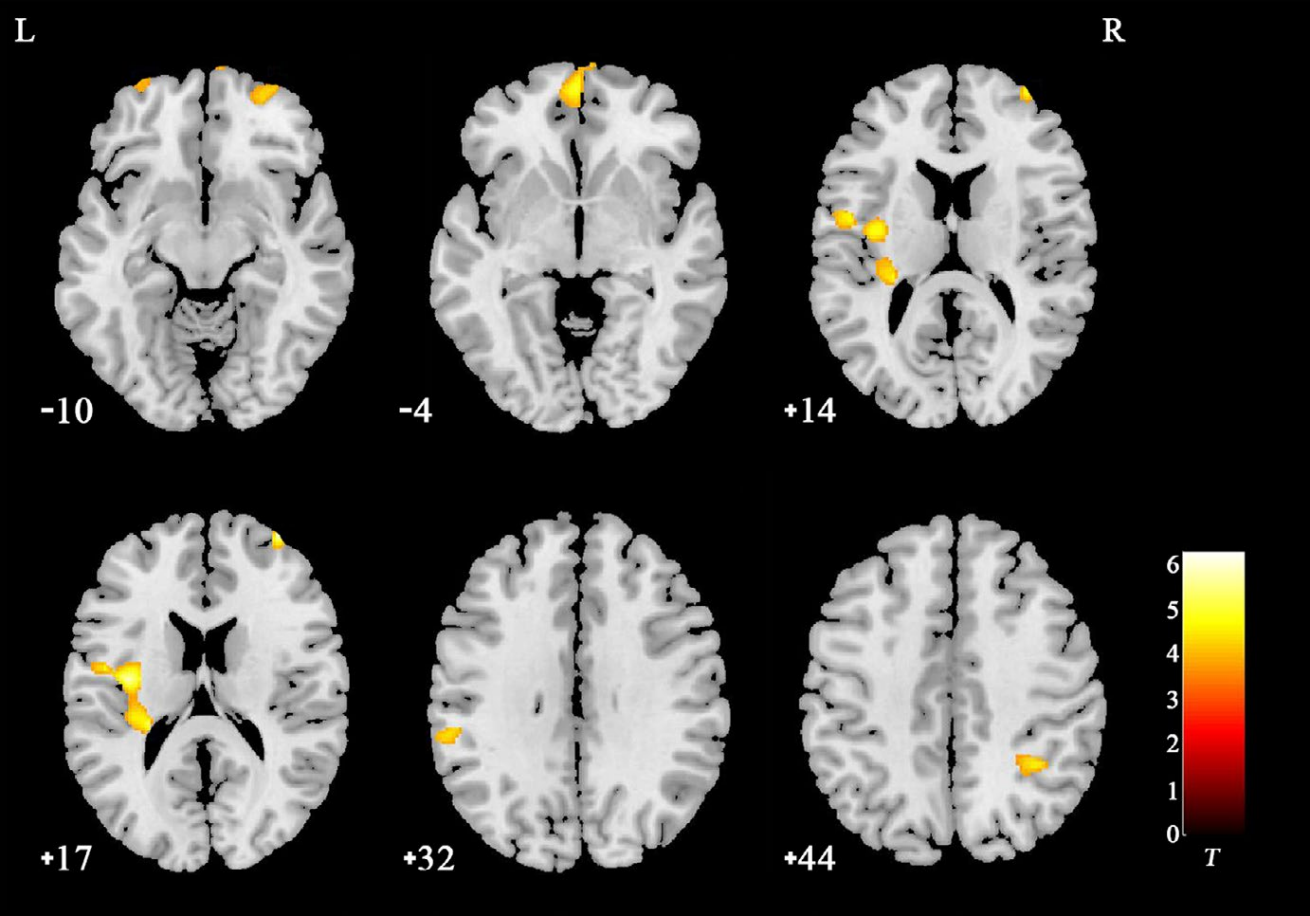
Reduced GMV in patients with VM compared to controls. Regions with significant reduction in VM were rendered onto the standard T1 template of Montreal Neurological Institute. Compared with NCs, the VM showed a significant decrease in GMV in the right middle frontal gyrus, the left orbital medial frontal gyrus, the right inferior parietal gyrus, the left supramarginal gyrus, the left posterior insular cortex, and the left operculum (*p* < .05, FDR corrected, with *k* > 100 voxels)

Several regions showed significant GMV reductions in VM patients compared with NCs; these regions included the middle frontal gyrus, orbital medial frontal gyrus, inferior parietal gyrus, supramarginal gyrus, posterior insula, and operculum (Table 2, Figure 1). No region showed a GMV increase in VM patients compared to NCs.

**Table 2:** Decreased GMV in various brain regions in VM patients

Brain regions	Peak MNI			Cluster voxels	*T*	*Z*	*p*
R							
Middle frontal gyrus	36	60	18	153	6.33	5.13	.028
Inferior parietal gyrus	35	−47	41	194	4.76	4.15	.036
L							
Orbital medial frontal gyrus	−2	60	−5	109	4.39	3.89	.040
Supramarginal gyrus	−60	−35	33	134	4.45	3.94	.036
Posterior insula	−37	−6	14	1,240	6.15	5.03	.028
Operculum	−38	−9	20	1,240	6.15	5.03	.028

Abbreviations: L, Left; R, right.

### 3.3 Correlation of clinical parameters with GMV

**Figure 2:**
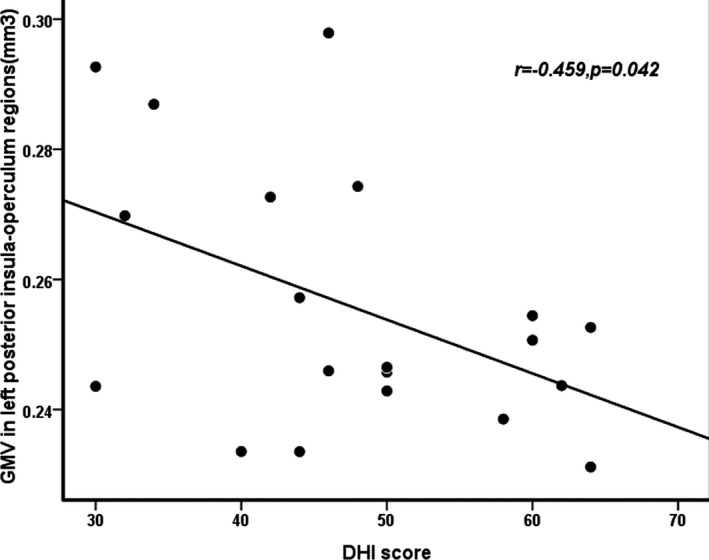
Correlation of GMV with DHI score. There was a negative correlation between GMV in the posterior insula–operculum regions and DHI scores in VM patients (*p* < .05)

The results of the correlation analysis showed that the DHI scores were negatively correlated with the volume of the left posterior insula–operculum regions (*p* = .042, *r* = −0.459; Figure 2).

## 4 DISCUSSION

In the present study, a significant decrease in GMV was observed in patients with VM compared to NCs. The main brain regions that showed a significant decrease in GMV were the PFC, the posterior insula–operculum regions, the inferior parietal gyrus, and the supramarginal gyrus. Correlation analysis revealed that the reduction of GMV in the posterior insula–operculum regions was negatively correlated with the severity of dizziness. There is evidence that abnormality of the central vestibular cortex is involved in the pathophysiology of VM; thus, the structural abnormalities in specific brain regions might contribute to clinical manifestations such as dizziness and vertigo. However, recurrent VM attacks might have cumulative effects on brain structure.

Our study found significantly decreased GMV mainly in the left hemisphere of patients with VM relative to NCs. These findings are inconsistent with several prior studies on the vestibular system (Dieterich & Brandt, 2015; Janzen et al., 2008; Schlindwein et al., 2008; zu Eulenburg et al., 2012). Previous studies showed that the vestibular sense is characterized by right‐hemisphere dominance in right‐handed people and left‐hemisphere dominance in left‐handed people. The factors that affect the underlying mechanisms of this lateral asymmetry are probably diverse. Such factors could involve the features of the heterogeneous samples included, such as patients with different unilateral symptoms, asymmetric onset, and differences in the vulnerability of the two cerebral hemispheres (Afridi et al., 2005b; Hougaard et al., 2015). Although all participants in the present study were right‐handed, most participants (14) with VM suffered from left unilateral headache, which may be a major determinant that contributes to the lateralization of vestibular structure. In the long term, repeated migraine attacks in the left hemisphere result in structural damage mainly to that hemisphere.

There are limited studies of gray matter morphology using MRI, especially VBM, to investigate the pathophysiological mechanisms of VM (Messina et al., 2017; Obermann et al., 2014; Wang et al., 2019). Using an ROI approach to VM patient detection based on previously reported structural findings in migraine and knowledge of the structures affected in vestibular disorders, Obermann and colleagues demonstrated a decreased GMV in the superior, inferior and middle (MT/V5) temporal gyrus, middle cingulate, dorsolateral prefrontal, insular, parietal, and occipital cortex, among other regions. In this study, the approaches were confined to a priori defined brain regions. Therefore, it is impossible to locate the widespread patterns of abnormalities across the brain in VM patients. In contrast, Messina and colleagues showed that VM patients had a selective increase in GMV in the frontal lobe, thalamus, temporal lobe, and occipital lobe when compared to NCs. These inconsistent results might be explained by demographic inconsistencies, such as white matter hyperintense lesions, in some VM patients who might affect the structural changes in the brain. Our study enrolled VM patients without white matter hyperintense lesions. Meanwhile, approaches in our analysis used voxel‐wise whole‐brain methods to avoid any unintentional bias by a priori hypotheses.

The striking finding of our study was the reduction of GMV in the posterior insula–operculum in VM patients. Previous reports have shown that the posterior insula received pain input from the spinothalamocortical circuit and is linked to the sensory‐discriminative aspects of pain processing (Garcia‐Larrea, 2012; Iannetti & Mouraux, 2010; Isnard, Magnin, Jung, Mauguiere, & Garcia‐Larrea, 2011; Mazzola, Faillenot, Barral, Mauguiere, & Peyron, 2012). Recently, positron emission tomography (PET) and functional MRI (fMRI) studies of VM have revealed abnormalities in the insula (Russo et al., 2014; Shin, Kim, Kim, & Kim, 2013). The other major finding of this study was the reduced GMV in the operculum, which was adjacent to the posterior insula. The posterior insular–opercular regions are believed to be contribute to pain transmission as the receiving areas of the spinothalamic system, which remains as the crucial part of the pain network (Frot, 2003; Garcia‐Larrea, 2012; Isnard et al., 2011; Mazzola et al., 2012). Therefore, our findings of GMV loss in these regions might reflect the important role of these transmission circuitry impairments in the pathophysiology of VM. Furthermore, GMV in the posterior insula–operculum regions was negatively correlated with the severity of vertigo in VM patients. From animal and human studies, the vestibular cortex includes the posterior insula, the superficial part of the temporoparietal junction and the superior temporal region, the sensorimotor cortex, the hippocampus, and many other structures of the parietal, frontal, and occipital lobes (Kahane, Hoffmann, Minotti, & Berthoz, 2003). A review of the vestibular system demonstrated the existence of a vestibular cortical core region in the parieto‐insular vestibular cortex (PIVC), which is located in the posterior insula, retroinsular region and parietal operculum (Ventre‐Dominey, 2014). However, it is not clear whether the vestibular cortical core region is a single area. Frank and colleagues, based on recent function and structural brain imaging studies (Billington & Smith, 2015; Frank & Greenlee, 2014; Frank, Sun, Forster, Tse, & Greenlee, 2016; Frank, Wirth, & Greenlee, 2016; Schindler & Bartels, 2018; Wirth, Frank, Greenlee, & Beer, 2018), have found some evidence for the existence of at least one other; this region has been named the posterior insular cortex area (PIC). The PIC is located in the retroinsular cortex posterior to the PIVC (Frank, Wirth, et al., 2016). The studies suggest that PIVC and PIC, although adjacent to each other, play different roles in the integration of visual and vestibular signals (Frank & Greenlee, 2014; Frank, Sun, et al., 2016; Frank, Wirth, et al., 2016). However, they are key regions of the cortical vestibular network. These areas are regarded as the core regions for receiving vestibular information and signal processing (Dieterich & Brandt, 2018; Ventre‐Dominey, 2014; zu Eulenburg et al., 2012). Lesion studies have shown that damaged insula area affects the perception of verticality or causes vertigo (Brandt, Botzel, Yousry, Dieterich, & Schulze, 1995; Halgren, Walter, Cherlow, & Crandall, 1978). Therefore, structural impairments in the posterior insula–operculum regions might result in central vestibular syndromes that manifest along with vertigo and dizziness.

The PFC is one of the most prominent areas associated with brain abnormalities in patients with migraine (Rocca et al., 2014; Schmitz et al., 2008; Schwedt & Dodick, 2009). Previous studies have suggested that the PFC plays a key role in connecting limbic and subcortical areas and is deemed to be associated with pain perception, modulation of pain, and cognitive and emotional variables (Apkarian, Baliki, & Geha, 2009; Apkarian, Bushnell, Treede, & Zubieta, 2005; Wiech, Ploner, & Tracey, 2008). Obermann and colleagues (Obermann et al., 2014) found decreased GMV in the PFC of VM patients compared to NCs using VBM methods. Our results also revealed that multiple areas within the PFC, including the middle frontal gyrus (MFG) and orbital medial frontal gyrus (OMFG), were altered in VM patients. One study suggested that structural PFC deficits, especially abnormalities in the MFG regions of the brain, might be closely related to the monitoring and temporal organization impairments in VM patients (Fassbender et al., 2004). According to a recent meta‐analysis of VBM results in patients with migraine, a significant GMV reduction in the middle gyrus was observed (Hu, Guo, Chen, & He, 2015). These findings are consistent with the above‐mentioned studies, suggesting that these areas were involved in cortical processing of vestibular and nociceptive information.

Additionally, several other regions with decreased GMV were observed, including the inferior parietal lobule and supramarginal gyrus. Alterations in the GMV of the inferior parietal lobule have been reported in many previous cerebral structural studies on migraine (Yu et al., 2016). Those studies showed that the cortical thickness of the inferior parietal lobe in the migraine patient group was significantly decreased compared with that in the healthy control group. Previous fMRI studies during vestibular stimulation in healthy subjects indicated a complex network of brain areas that are involved in the equilibrium and spatial navigation (Bottini et al., 2001). Among these areas, the inferior parietal lobule has been involved in maintaining attention when working toward the current task goals and responding to the salient new information or alerting stimuli in the environment (Singh‐Curry & Husain, 2009). A previous study showed activation of the inferior parietal lobule in VM patients (Teggi et al., 2016). The inferior parietal lobule mainly functions in the pain response and the sensing of temperature and pressure. This region is also part of the multisensory vestibular cortical network (Dieterich & Brandt, 2008). Our findings in VM patients suggested that it might be associated with self‐adaptation of the nervous system and global dysfunction of sensory integration and memory processes during the interictal phase. The supramarginal gyrus is involved in a variety of cognitive functions such as visual attention and imagery (Hanakawa, Dimyan, & Hallett, 2008; Hartwigsen et al., 2012). Structural abnormalities of the supramarginal gyrus have also been demonstrated in patients with migraine, including migraine without aura and migraine with aura (Rocca et al., 2014). Interestingly, Ferraro et al demonstrated that medication overuse in headache patients has a reduced pain‐related activity of the supramarginal gyrus (Ferraro et al., 2012). The supramarginal gyrus might be involved in general pain processing or in pain response.

### 4.1 Limitations

Several limitations of the current study may require some consideration. First, the number of VM patients is relatively small, and the necessity for additional study in examining large samples may help elucidate the apparent volumetric changes in VM. Second, preventive therapies that might influence the brain morphological results could not be excluded. Third, migraine subtypes such as migraine without aura (MWoA) and migraine with aura (MWA) were not identified. Finally, our study merely utilized the methodology for examining GMV in VM patients. Future studies might combine structural and functional analyses to help us better understand the pathophysiology of VM.

## 5 CONCLUSIONS

In summary, the current study showed alterations in the vestibular cortex of patients with VM. Some of these findings, particularly in the posterior insula–operculum, involve vestibular cortical core regions that play a pathophysiological role in patients with VM.

## CONFLICT OF INTEREST

The authors declare no conflict of interests.

## AUTHOR CONTRIBUTIONS

Xia Zhe drafted the manuscript, study concept or design, and statistical analysis. Li Chen undertook clinical parameters assessments. Fuxia Bai, Ze Zou, Xin Zhang, and Weibo Chen provided technical support. Jie Gao, Min Tang, Dongsheng Zhang, Xuejiao Yan, and Xiaoyan Lei acquisition of data. Xiaoling Zhang study supervision or coordination. All authors read and approved the final manuscript.

## Data Availability

The data that support the findings of this study are available on requests from the corresponding author.
